# Employment and work ability in individuals living with rare diseases: a systematic literature review

**DOI:** 10.1186/s13023-025-03691-7

**Published:** 2025-04-23

**Authors:** Nicolas Bougas, Terhi Kangas, Katrien Vanthomme, Jose Joaquín Mira Solves, Gaël Brulé, Helene Mellerio, Hadewijch Vandenheede, Agnes Dumas

**Affiliations:** 1 Université Paris Cité, Inserm, ECEVE, UMR 1123, 10 Avenue de Verdun, 75010 Paris, France; 2 Department of Sociology, BRISPO, Vrije Universiteit Brussel, Pleinlaan 2, 1050 Brussels, Belgium; 3 Department of Public Health and Primary Care, Ghent University, Sint-Pietersnieuwstraat 33, Ghent, Belgium; 4 Alicante-Sant Joan Healthcare District, FISABIO, Alicante (Spain), Miguel Hernandez University, Elche, Spain; 5 Geneva School of Health Sciences, 47 Avenue de Champel, 1206 Geneva, Switzerland; 6 APHP - Assistance Publique-Hôpitaux de Paris, Robert Debré Hospital, AD’venir, Adolescent Medicine Unit, 48 Boulevard Sérurier, 75019 Paris, France; 7 French Clinical Research Group in Adolescent Medicine and Health, 97 Boulevard de Port-Royal, 75014 Paris, France; 8 INSERM, Aix Marseille Univ, IRD, ISSPAM, SESSTIM, Sciences Economiques & Sociales de La Santé & Traitement de L’Information Médicale, Equipe CALIPSO, 27 Boulevard Jean Moulin, 13005 Marseille, France

**Keywords:** Rare diseases, Absenteeism, Presenteeism, Work, Employment, Quality of life

## Abstract

**Background:**

The socioeconomic impact of rare diseases has been mostly studied at the macrolevel, but evidence at the microlevel is lacking, which overshadows health-related social inequalities affecting people with rare diseases, namely, health selection effects.

**Aim:**

This study presents an overview of employment and work ability in individuals living with rare diseases, two factors related to health selection effects.

**Methods:**

A systematic literature review was conducted using the PRISMA checklist. Three electronic databases, PubMed, Embase, and Web of Science, were searched from 2013 to 2023. Eligible studies needed to investigate at least one work-related outcome measuring employment or work ability in individuals living with rare diseases and to compare it with a control group. Indeed, including only studies with matched or standardized control groups is essential for ensuring the reliability and validity of research findings.

**Results:**

Of the 7,694 abstracts identified, 44 studies, including 34 rare diseases, met the inclusion criteria. Administrative databases were used to collect work-related data in 48% of the studies, and 73% of the studies employed matching methods for comparison. Overall, 52% of the studies focused solely on employment, 14% focused solely on work ability and 34% included both categories. Individuals with rare diseases were less likely to be employed or more likely to be unemployed than controls in 68% of the studies and 87% of the studies reported that individuals with rare diseases were more likely to be work disabled. Regarding work ability, 90% of the studies reported more missed work time in cases than in controls, and more perceived impairment at work was found in 100% of the studies.

**Discussion/conclusion:**

These results show that individuals with rare diseases tend to have poor work outcomes, but methodological limitations hamper the understanding of health selection effects. Implications for future research and policy-making are discussed.

**Supplementary Information:**

The online version contains supplementary material available at 10.1186/s13023-025-03691-7.

## Introduction

A disease is considered rare when it affects fewer than 200,000 individuals in the United States or less than 1 in 2000 people in Europe [1, 2]. While a single rare disease may impact a small number of patients, collectively, these conditions are estimated to impact 3.5–5.9% of the global population, affecting approximately 263–446 million individuals worldwide [3].

Advancements in several rare disease treatments have significantly improved patients’ prospects by providing better symptom relief, slowing disease progression, and potentially introducing new curative options expanding life expectancy [4]. Medical progress has redirected the research focus towards the health-related quality of life of individuals living with rare diseases [5] and towards the socioeconomic burden or impact of rare diseases [6, 7]. However, the majority of studies evaluating the impact of rare diseases are conducted at the macro level using cost-of-illnesses approaches, with an emphasis on economic features [7]. To date, there is no overview of the results of these macrolevel studies [6, 7], while a scoping review recently shed light on work participation in adults with rare genetic diseases and the factors associated with work participation [8]. However, there remains a gap in understanding the microlevel impact on individuals, notably the social impact of rare diseases and the so-called health selection effects.

The health selection effect refers to the idea that health status, e.g., having a rare disease, affects the social mobility of unhealthy individuals, who tend to drift down the social scale or to reach a lower socioeconomic position than expected considering their socioeconomic background [9]. Health selection effects are particularly at stake for individuals affected by diseases with childhood onset because of the possible difficulties experienced in education or work throughout life. Although more than 70% of rare diseases are either genetic or have a paediatric onset [3], health selection effects have been little studied in rare diseases compared to frequent chronic diseases with a childhood onset [10]. Thanks to medical progress, an increasing proportion of children with rare diseases are reaching adulthood and working age. Even if the effects of rare diseases on health-related quality of life are varied, a significant proportion of rare diseases can result in cognitive or mobility impairment, and a high proportion are degenerative and life-threatening [11] and/or require a considerable follow-up time in scarce expert centres, as multiple organ systems are often affected [12]. Despite variations in symptoms, rare diseases patients are increasingly recognized as a population sharing common psychological and social vulnerabilities [13, 14], and a global overview of work-related challenges faced by adults living with rare diseases can be of particular interest for policy makers, clinicians and researchers. In addition, it is important to recognize the work outcomes of adults with rare diseases, as they may differ from those of adults with more common chronic conditions, who are typically older. These differences arise due to age-specific work-related challenges, since the impact of health on work life varies by age, with older individuals dealing with retirement concerns and younger ones facing employment entry barriers. Understanding these unique pathways can inform better interventions and policies. Currently, there is a lack of robust evidence in this field due to variations in research designs and methodologies, as outlined by a scoping review on work participation in adults with rare genetic disease [8]. For instance, an average work participation rate of 55% was found, but this estimate was based on studies involving young participants who may be still in school that may skew the results. However, being a scoping review, the study provides an indicative estimate rather than a scientifically robust figure. The lack of control groups matched by age and sex, coupled with inconsistent definitions of outcomes, complicates the calculation of reliable rates. To effectively support healthcare professionals and decision-makers, it is crucial to synthesize literature based on well-designed studies. This study aimed to bridge this gap by presenting a comprehensive overview of the employment and work ability of individuals living with rare diseases, two factors related to health selection effects holding particular significance for their role in social integration and the overall quality of life of individuals [15, 16]. Moreover, this study aimed to understand if people with rare diseases differ in terms of employment and work ability compared to matched controls. Including only studies with matched or standardized control groups is essential for ensuring the reliability and validity of research findings. It can eliminate confounding variables and help isolate the specific impact of the disease on outcomes like employment; it may improve comparability and thus support evidence-based decision-making.

## Methods

We used the PRISMA (Preferred Reporting Items for Systematic Reviews and Meta-Analyses Statement) checklist to ensure thoroughness in conducting and reporting the systematic review [17]. Three electronic databases—PubMed, Web of Science, and Embase—were used to conduct the systematic review. The protocol of the study was deposited on Prospero (CRD42023474673).

### Search strategy

The search query consisted of two parts linked by the Boolean operator “AND”. In the first part, we included general terms describing rare diseases (e.g., “rare disease”) and the names and synonyms of the 695 most prevalent rare diseases, i.e., those with a point prevalence or annual incidence > 1/100,000 in Europe or worldwide registered in the Orphanet database (http://www.orphadata.org/cgi-bin/epidemio.html), linked using the Boolean operator “OR”. In the second part, we included work-related terms connected using the Boolean operator “OR”. The terms of both parts (names of rare diseases and work-related terms) are displayed in Supplemental Table S1. To refine the search, the Boolean operator “NOT” was used to exclude specific keywords from titles, e.g., studies focusing on treating rare diseases or those investigating the impact of the COVID-19 pandemic on individuals with rare diseases (Supplemental Table S1). Our search was limited to original research articles published in English between January 2013 and May 2023. This specific timeframe was chosen to ensure the feasibility of conducting a comprehensive systematic literature review. The customized search query was uniformly applied across the three selected databases (Supplemental Table S1). We chose two databases that focus on biomedical academic literature (PubMed & Embase) and one that covers more scientific fields (Web of Science) to ensure the thoroughness of this systematic review.

### Eligibility and exclusion criteria

Overall, eligible studies needed to investigate at least one work-related outcome measuring employment or work ability in individuals living with rare diseases by comparing outcomes with a control group.

The studies had to be observational and include adult subjects with rare diseases. Studies on childhood cancers or traumatic brain injuries were excluded given the considerable amount of literature published on those conditions [18, 19].

Comparative analyses employing suitable statistical methods (e.g., chi-square tests, t tests, or odds ratios) were essential to compare work-related outcomes between patients with a rare disease and their respective controls. Studies with no matched or standardized control group or without any adjustment in the statistical analyses were excluded to avoid bias in the comparison between cases and controls.

Studies comparing work-related outcomes before and after the diagnosis of a rare disease within the same group of patients were excluded unless a control group was included. Similarly, studies that compared work-related outcomes among different types, severity levels, grades, or symptoms of rare diseases or different treatments were also excluded.

### Study selection and data extraction

**Fig. 1:**
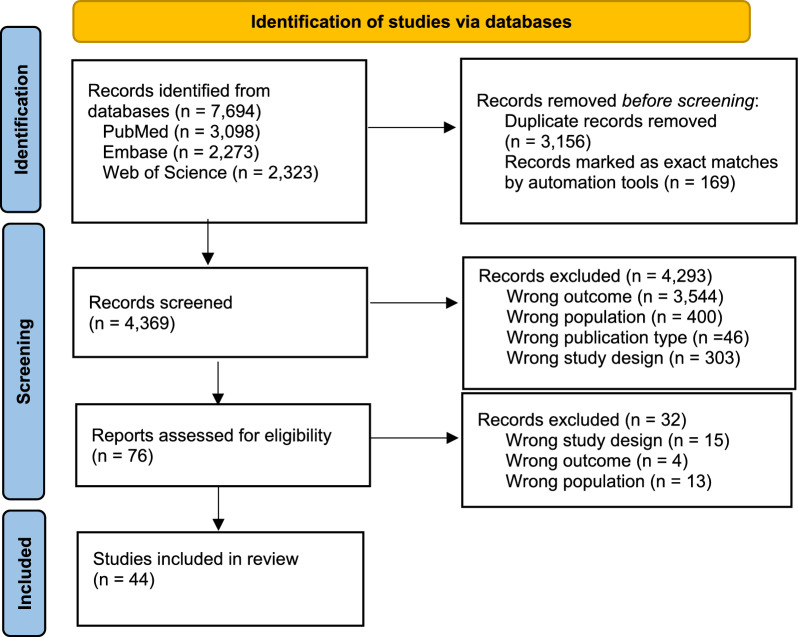
PRISMA flow diagram for new systematic reviews [17]

Of the 7694 abstracts identified through the databases and imported into the Rayyan software [20], 3325 duplicates were removed, and 4,369 were screened. Of these, 4293 were excluded, mainly because they did not investigate work-related outcomes (wrong outcome, n = 3544); reported work-related outcomes in an excluded population such as individuals with traumatic brain injury or survivors of childhood cancers (wrong population, *n* = 400); or did not include an appropriate design, such as a qualitative design or no control group (wrong study design, *n* = 303). Full-text screening was conducted for 76 reports, 44 of which met the eligibility criteria and were included in the review (Fig. 1).

Two reviewers (NB, TK) independently conducted the selection process for eligible studies. The authors were blinded for each other’s decisions. Any disparities in the extracted data were resolved through discussion. In cases where discrepancies persisted, a third independent reviewer (AD) was consulted to resolve the remaining conflicts. The risk of bias and the methodological quality were independently assessed by the two reviewers using a checklist adapted from the Newcastle‒Ottawa Quality Assessment Scale (NOS) for case‒control studies to evaluate the reliability and validity of the included studies [21]. The scale was adapted for one item that initially assesses the ascertainment of exposures in case‒control studies: this item was changed to specifically assess the ascertainment of work-related outcomes. Descriptive data regarding the timing of onset and the type of impairment related to the rare diseases of the included studies were collected by web searches if such information could not be retrieved from the manuscripts. The analysis was made using an a priori extraction grid. The results reported in each study were detailed by two reviewers (NB, TK) in two different grids for the two main categories of outcomes (employment, work ability). One reviewer (AD) verified the accuracy of the extraction grids. A meta-analysis was not conducted due to the heterogeneity of the included studies, particularly in terms of outcome measures, which limited the feasibility of pooling data in a statistically meaningful way. Thus, we focused on the significancy of results showing a poorer situation for individuals living with rare diseases: these significant results were considered per study and per type of outcome since one study could include several types of outcomes.

## Results

### Description of the included studies

The 44 studies included in the review focused on 34 different rare diseases [22–65]. The most studied diseases were Systemic Lupus Erythematosus (SLE) (5 studies), narcolepsy, and Turner syndrome (3 studies each). Of these 34 diseases, 22 may exhibit cognitive or mobility impairment to varying degrees: 12 have a potential cognitive impact, while 18 exhibited a possibility of impacting mobility. Of the 34 diseases included, 5 had congenital onset, 3 had only childhood onset, and 26 had either childhood or adulthood onset.

Table 1 presents the characteristics of the 44 included studies, and Supplemental Table S2 provides the details for each study, including the NOS score. Almost one-third of the studies (32%) had a score lower than 6 (Supplemental Table S2). The sample size ranged from 31 to 9312 participants. On average, the mean age at inclusion was 42.8 years, and 63% of the participants were women. Regionally, 64% of studies were conducted in Europe, and 30% in North America. In 48% of the 44 studies, cases were identified using administrative databases (i.e. using national healthcare registries from the Nordic countries in 25% of studies or large health claims databases in 18% of studies, which came mainly from the USA). Other studies used existing national or local registries of rare diseases (16%) or relied on a retrospective study design using hospital records from one (14%) or several (16%) centres. The remaining studies used national health surveys or large biobanks (7%). Work-related outcomes were extracted for both cases and controls from administrative data in 48% of studies and from questionnaires in 36%; the remaining studies used questionnaires for cases and national statistics for controls (16%). All studies included matching or adjustment for age and sex. Overall, 32 studies (73%) were based on matching methods for comparison (Table 1).

**Table 1:** Characteristics of included studies (*N* = 44)

Characteristics		
Country	*N*	%
Europe	28	63.6
North America	13	29.5
Other	3	6.8
Number of cases		
< 100	9	20.5
100–299	13	29.5
300–1000	13	29.5
> 1000	9	20.5
Mean age of cases at the time of the study		
< 30 years old	5	11.4
30–50 years old	26	59.1
> 50 years old	13	29.5
Recruitment of cases		
National healthcare registry	11	25.0
Large health claims databases	8	18.2
Multicenter retrospective study	7	15.9
Single center retrospective study	6	13.6
Single center prospective registry	3	6.8
National population-based health surveys or cohorts	3	6.8
Local healthcare registry	2	4.5
National rare disease registry	2	4.5
Multicenter prospective RD registry	2	4.5
Recruitment of controls		
Local/National administrative registry	19	43.2
Large health claims databases	8	18.2
National statistics	7	15.9
National population-based health surveys	6	13.6
Siblings, friends	2	4.5
Not stated	2	4.5
Source of work-related data in cases		
Administrative data	21	47.7
Questionnaire	23	52.3
Source of work-related data in controls		
Administrative data	23	52.3
Questionnaire	14	31.8
National statistics	7	15.9
Method of comparison with controls		
Matching	33	75.0
Standardization	8	18.2
Adjustment or subgroup analyses	3	6.8
Quality assessment score (Newcastle–Ottawa)		
< 6	14	31.8
6	16	36.4
> 6	14	31.8

Regarding the medical characteristics of controls, 30 studies (68%) reported that the controls were free of the rare disease under investigation. Of these 30 studies, 12 studies used additional methods: 4 studies excluded specified comorbidities [42, 43, 64, 65] but two of them did not provide information on the health ascertainment of controls [42, 43]; 4 studies matched patients and controls on comorbidities using the Charlson Comorbidity Index (CCI) [39, 49, 60, 63], one study used the CCI and additional comorbidities for the matching [29], one study used the CCI for matching and also excluded other specified conditions associated with the rare disease investigated [56], and one study used the CCI for subgroup analyses [54]; in addition, controls were self-reported as healthy in one study [25]. The 14 studies with no matching or adjustment on medical characteristics (32%) mainly used reference data from the general population (Supplemental Table S2).

Overall, 23 studies (52%) matched cases and controls using social variables: 15 studies only used an ecological variable (place of residence in 14 studies [22, 24, 28, 34–36, 44, 46, 48, 50, 51, 55, 56, 61] and place of birth in one study [62]), six studies included individual-level variables such as income, education, employment status, or race [23, 45, 52, 60, 63, 65] and two studies included type of insurance [27, 31]. In addition, four studies (9%) used other methods than matching to consider the social features of controls: two studies used siblings or friends to recruit controls [25, 26], and two studies adjusted the analysis using socioeconomic variables [53, 57]. The social characteristics of participants were not controlled in the 17 remaining studies (39%) (Supplemental Table S2).

The 44 studies included 19 different types of outcomes, which are displayed in Table 2 for a global overview of the results. These 19 outcomes were categorized into two main categories: 1) employment status, such as whether individuals are employed, unemployed, or receive a disability pension, and 2) work ability, which can be studied from two perspectives, namely, absenteeism (e.g., missed work time because of health) and presenteeism (e.g., self-perceived impairment at work). A few studies based on administrative data also included a third category of outcome identified as “work loss”, combining the two main categories of outcomes (employment status and work ability), as they merged insurance data on disability pensions and sick leave benefits into a single measure (Table 2). Overall, 52% of the studies (n = 23) focused solely on employment, 14% (n = 6) focused solely on work ability and 34% (n = 15) included both categories. Studies including outcomes on work ability or work loss were more likely to rely on administrative data (48% of the 21 studies) than studies focusing exclusively on employment outcomes (26% of the 23 studies) (Supplemental Table S2).

**Table 2:** Number of significant results showing a poorer situation for individuals living with a rare disease (cases) per results and per studies

Main categories of outcomes	Sub-categories of outcome	Detailed types of outcomes (*N* = 117)	N. of significant results showing a poorer situation for cases* (*N* = 84/117)	N. of studies with at least one significant result showing a poorer situation for cases (*N* = 34/44)	
**Employment status** (69 results in 38 studies)	Employment (29 results in 22 studies)	Being active (Y/N; Yes: whether employed or seeking work), *N* = 1	0	15	
		Being employed (Y/N; Yes: whether paid or not), *N* = 16	11		
		Being in paid work (Y/N; Yes: receiving income from work), *N* = 6	3		
		Being full time employed (Y/N), *N* = 2	1		
		Being part time employed (Y/N), *N* = 4	2		
	Unemployment (21 results in 15 studies)	Being inactive (Y/N; Yes: whether disabled, student, housewife, retired, other), *N* = 4	3	10	
		Being unemployed (Y/N; Yes: seeking work or unemployed because of health), *N* = 5	1		
		Seeking work (Y/N), *N* = 5	3		
		Being unemployed because of health, receiving a pension or not (Y/N), *N* = 7	6		
	Disability (19 results in 16 studies)	Being disabled, receiving a pension or not (Y/N; Yes: short- or long-term disabled), *N* = 3	2	14	
		Receiving a disability pension (Y/N; Yes: short- or long-term disability pension), *N* = 10	9		
		Number of days with a short- or long-term disability pension, *N* = 6	5		
**Work ability or Work loss in employed individuals** (47 results in 21 studies, including 41 results in 20 studies for Work ability and 6 results in 5 studies for Work loss)	**Work ability:** Absenteeism (29 results in 20 studies)	Reporting missed work time because of health problems (Y/N (Yes: at least 1 h or 1 day of missed work time) or proportion of missed work time (% of hours or days over a given period)), *N* = 13	8	18	
		Being on sick leave (Y/N or number of days), *N* = 11	9		
		Being on long-term sick leave (Y/N; Yes: > 2 weeks sick-leave or proportion of time over a given period), *N* = 5	5		
	**Work ability:** Presenteeism (12 results in 6 studies)	Feeling impaired at work because of health (Y/N), *N* = 4	3	6	
		Proportion of self-perceived impairment (Proportion of working time feeling impaired at work because of health), *N* = 6	5		
		Proportion of self-perceived work capacity/productivity (Physical or mental, usually represented with a score), *N* = 2	2		
	**Work loss** Combination of disability and absenteeism (6 results in 5 studies)	Work loss because of health problems or disability (Reported work loss (Y/N) or proportion of work loss, *N* = 6	5	5	

^*^Since a study could report different results ensuing from diverse types of outcomes, a total of 118 results extracted from the 44 studies are reported in Table 2

Abbreviations. Y/N: Yes/No, N.: Number

### 1) Narrative synthesis of the results: Employment status

The results regarding employment status are detailed for each study in Table 3, while Table 2 shows a global overview of the results for each type of outcome. The outcomes related to employment status were explored across 38 studies and 31 rare diseases, encompassing a total of 69 different results on i) employment (n = 29), ii) unemployment (n = 21), and iii) disability (n = 19) (Table 2). A poorer employment status for individuals living with rare diseases was found in 46 out of 69 results (Table 2), with at least one significant result in 31 out of 38 studies (Table 3).

**Table 3:** Results regarding employment status

Ref	Region	Rare disease(s)	Timing of onset	Possible cognitive impairment	Possible mobility impairment	Age*	Significant results showing a poorer outcome for individuals with a rare disease (cases)	Non-significant (NS) results or opposite result
[21]	E	Acute hepatic porphyria	C/A	No	No	31	Cases more likely to receive a disability pension	_
[22]	E	Sarcoidosis	C/A	Yes	Yes	30–39	Cases less likely to be in paid work	_
[23]	O	Childhood onset multiple pituitary hormone deficiencies (COMPHD)	C	Yes	Yes	30	Cases less likely to be employed Cases more likely to be seeking work Cases more likely to be unemployed because of health	_
[24]	NA	Systemic lupus erythematosus	C/A	Yes	Yes	18–34	Cases less likely to be full time employed Cases more likely to be unemployed because of health	Being in part time employment (NS) Seeking work (NS)
[25]	NA	Narcolepsy	C/A	No	No	41	Cases have more days of short-term disability	_
[27]	NA	Dermatomyositis, Polymyositis	C/A	No	Yes	49	_	Number of disability days (NS)
[28]	NA	Idiopathic lung disease, Idiopathic nonspecific interstitial pneumonia, Chronic hyper-sensitivity pneumonitis	C/A	No	No	69	Cases less likely to be employed	_
[29]	NA	Systemic sclerosis	C/A	No	Yes	58	Cases having more disability days	_
[30]	E	Hemophilia	Birth	No	No	41	Cases less likely to be employed Cases more likely to be seeking work Cases more likely to be inactive Cases more likely to be in part time employment	_
[31]	NA	Fibrotic CTD-ILD	C/A	No	No	60	Cases less likely to be employed	_
[32]	NA	Juvenile idiopathic arthritis	C	No	Yes	19	_	Being employed (NS) Being unemployed (NS) Being disabled (NS)
[33]	E	Sjögren's syndrome	C/A	Yes	Yes	46	Cases more likely to receive a disability pension 2 y. after diagnosis	Receiving a disability pension at diagnosis or 1 y. after (NS)
[34]	E	Sjögren's syndrome	C/A	Yes	Yes	63	_	Being unemployed (NS)
[35]	E	Juvenile idiopathic arthritis	C	No	Yes	34	_	Being unemployed (NS)
[36]	E	Turner Syndrome	Birth	Yes	No	28	Cases more likely to be unemployed because of health	Being in paid work (NS) Seeking work (NS)
		Systemic lupus erythematosus	C/A	Yes	Yes	55	Cases less likely to be in paid work Cases more likely to be unemployed because of health	_
[38]	NA	Systemic sclerosis	C/A	Yes	Yes	55	_	Being in paid work (NS) Unemployment because of health (NS)
		Sjögren's syndrome	C/A	Yes	Yes	55	Cases more likely to be unemployed because of health	Being in paid work (NS)
[39]	E	Systemic lupus erythematosus	C/A	Yes	Yes	42	Cases more likely to receive a disability pension	_
[41]	O	Retinitis Pigmentosa	C/A	No	Yes	44	Cases less likely to be employed	_
[42]	E	Idiopathic/genetic generalized epilepsies (IGEs)	C	Yes	Yes	35	Cases more likely to be inactive	
[43]	E	Myasthenia gravis	C/A	No	Yes	43	Cases more likely to be inactive	_
[44]	E	Systemic lupus erythematosus	C/A	Yes	Yes	33	Cases less likely to be employed	_
[45]	E	Systemic lupus erythematosus	C/A	Yes	Yes	33	Cases more likely to be in part time employment Cases more likely to be disabled	_
[46]	E	Turner Syndrome	Birth	Yes	No	41	Cases more likely to receive a disability pension	Being employed (NS) Being in part time work (NS)
[49]	E	Narcolepsy	C/A	No	No	20	Cases more likely to be unemployed	_
[50]	NA	Acromegaly	C/A	No	Yes	48	Cases have more short-term disability days	_
[51]	E	Hemophilia	Birth	No	No	16–44	Cases less likely to be employed	_
[52]	E	Atrial Septal Defect	Birth	No	No	30	Cases less likely to be employed Cases more likely to be seeking work Cases more likely to receive a disability pension	_
[53]	E	Pulmonary Arterial Hypertension	C/A	No	No	62	Cases less likely to be employed Cases more likely to receive a disability pension Cases have more disability days	_
[54]	NA	Non-infectious posterior uveitis, Panuveitis	C/A	No	Yes	48	Cases more likely to receive a long-term disability pension Cases have more disability days	_
[55]	E	Crouzon Syndrome	Birth	Yes	No	35	_	Being employed (NS) Being full-time employed (NS) Being unemployed (NS)
[56]	NA	Turner Syndrome	Birth	Yes	No	38	_	Cases more likely to be employed (opposite result)_
[57]	E	Juvenile Dermatomyositis	C	Yes	No	21	Cases less likely to be employed	_
[58]	NA	Narcolepsy	C/A	No	No	47	Cases more likely to be long-term disabled	Being active (NS)
[59]	E	Hypersomnia	C/A	No	No	50–59	Cases less likely to be in paid work	_
[60]	E	Congenital adrenal hyperplasia	Birth	No	No	20–50	Cases more likely to receive a disability pension	Being employed (NS)
[61]	E	Bladder pain	C/A	No	No	48	_	Being inactive (NS)
[62]	E	Meningococcal meningitis	C/A	Yes	Yes	35	Cases less likely to be employed Cases more likely to receive a disability pension	_
[63]	E	Aneurysmal Subarachnoid Haemorrhage	C/A	Yes	No	58	Cases more likely to be unemployed because of health	_

Abbreviations. Ref. Reference, NA: North America, E: Europe, O: other, C: Childhood, C/A: Childhood/Adulthood, y.: year

^*^Mean age at study or most prevalent age group of cases

***i) Employment:*** At least one of the work-related outcomes was poorer for individuals with rare diseases than for controls in 68% of studies (n = 15/22). In most studies, employment was assessed as “being employed” (whether paid or not, whether working full-time or part-time). The results regarding paid work or work time (working part-time or full-time) were more contrasted than those for “being employed” (50% versus 68% showed a poorer situation for cases respectively) (Table 2). In contrast, one opposite result was detected, with significantly more employed patients with Turner syndrome than controls [58].

***ii) Unemployment***: There was significantly more unemployment in rare disease groups than in controls in 68% of studies (n = 10/15). The results were very likely to show a poorer situation for patients when “unemployment because of health” was the outcome of interest (6/7 results were significant) compared to results based solely on “unemployment” (1/5 result being significant) (Table 2).

***iii) Disability***: There were significant differences between cases and controls in 87% of studies (n = 14/16) assessing disability, showing that patients were more likely to, for example, be on a disability pension, be work disabled, or have a greater number of days of disability compared to their controls (Table 2). Notably, significant differences in disability might not immediately manifest but might develop over time, as observed with longitudinal follow-up [35].

In summary, individuals with rare diseases generally exhibited significantly lower employment rates and greater disability rates than controls, with less clear patterns found regarding “unemployment” (i.e., when the outcome did not specifically target “health-related unemployment”). In addition, all 5 studies on SLE reported significantly worse employment situations for patients [26, 40, 41, 46, 47]. In contrast, childhood-onset Juvenile Idiopathic Arthritis (JIA) did not significantly differ in any of the outcomes studied, in terms of employment, unemployment, or work disability [34, 37]. Notably, for Turner syndrome patients, while no significant difference was found in unemployment [38] or employment rates [38, 48], significantly more cases were observed to be permanently sick or disabled [38] or on disability pensions than in controls [48] (Table 3).

### 2) Narrative synthesis of the results: work ability and work loss

Table 4 presents detailed results related to work ability and work loss per study, while Table 2 shows a global overview of the results per type of outcome. A total of 47 results were explored in 21 studies assessing i) absenteeism (29 results), ii) presenteeism (12 results) and iii) work loss (6 results) (Table 2). Overall, a significantly poorer situation regarding work ability or work loss was found for individuals with rare diseases in 95% of the studies (n = 20/21) (Table 4).

**Table 4:** Results regarding work ability and work loss

Ref	Region	Rare Disease(s)	Timing of Onset	Possible Cognitive Impairment	Possible Mobility Impairment	Age*	Time Frame	Significant results showing a poorer outcome for individuals with a rare disease (cases)	Non-significant (NS) results
[20]	E	Myasthenia gravis	C/A	No	Yes	64	Unknown	Cases more often on sick leave (Y/N)	_
								Cases more often received sickness benefit (Y/N)	
[21]	E	Acute hepatic porphyria	C/A	No	No	31	FU period	Cases with more long-term sick leave (> 17 days)	_
[24]	NA	Systemic lupus erythematosus	C/A	Yes	Yes	18–34	Last month	Cases with a higher number of sick leave days	_
								Cases with less self-perceived work productivity	
[26]	E	Sarcoidosis	C/A	Yes	Yes	43	Various	Cases with a higher number of days of work loss 5 y. after diagnosis	_
[27]	NA	Dermatomyositis, Polymyositis	C/A	No	Yes	50	Year followin	Cases with a higher number of sick leave days	_
[29]	NA	Systemic sclerosis	C/A	No	Yes	58	Over a year	Cases with a higher proportion of missed work time	_
								Cases with more work loss	
[33]	E	Sjögren's syndrome	C/A	Yes	Yes	46	Various	Cases more often on sick leave (Y/N) 1 and 2 y. after diagnosis	Work loss 1 y. after diagnosis (Y/N)
								Cases with more work loss (Y/N) 2 y. after diagnosis	
[37]	E	Non-tuberculous mycobacterial pulmonary disease	C/A	No	No	50	FU period	Cases with a higher number of sick leave days	_
		Systemic lupus erythematosus	C/A	Yes	Yes	55	Last	Cases more often missed > 1 h of work time	Number of hours of missed work time
							week	Cases with a higher proportion of perceived impairment at work	Felt impaired at work (Y/N) > 1 h missed
[38]	NA	Systemic sclerosis	C/A	Yes	Yes	55	Last week	Cases more often felt impaired at work	Number of hours of missed work time
									Proportion of perceived impairment at work
		Sjögren's syndrome	C/A	Yes	Yes	55	Last week	Cases more often missed > 1 h of work time	Number of hours of missed work time
								Cases more often felt impaired at work	
								Cases with a higher proportion of perceived impairment	
[40]	O	Familial Mediterranean fever	C/A	No	Yes	33	Last week	Cases with a higher number of hours of missed work time	Missed > 1 h of work time
								Cases with a higher proportion of missed work hours	
								Cases more often felt impaired at work	
								Cases with a higher proportion of perceived impairment	
[43]	E	Myasthenia gravis	C/A	No	Yes	43	1 and 2 years after diagnosis	Cases more likely to have a long-term sick leave	
								(> 9 weeks) 1 and 2 y. after diagnosis	
[44]	E	Systemic lupus erythematosus	C/A	Yes	Yes	33	Last year	Cases with a higher number of sick leave days	_
								Cases with a lower perceived work capacity regarding physical or mental demands	
[47]	E	Bronchiectasis	C/A	No	Yes	59	FU period	_	Number of sick leave days
[48]	NA	Pulmonary Arterial Hypertension	C/A	No	No	53	Over the FU period	Cases with a higher number of missed work days (reported by the employer)	_
[50]	NA	Acromegaly	C/A	No	Yes	48	FU period	_	Mean number of sick leave days
[52]	E	Atrial Septal Defect	Birth	No	No	30	FU period	Cases with a higher proportion of time on long-term sick leave	_
[53]	E	Pulmonary Arterial Hypertension	C/A	No	No	62	Year after diagnosis	Cases with a higher number of sick leave days	_
[54]	NA	Non-infectious posterior uveitis, Panuveitis	C/A	No	Yes	48	FU period	Cases with a higher number of days of sick leave	_
								Cases with a higher number of days of work loss	
[58]	NA	Narcolepsy	C/A	No	No	47	Last week	Cases with a higher proportion of missed work time	_
								Cases with a higher proportion of perceived impairment	
[60]	E	Congenital adrenal hyperplasia	Birth	No	No	20–50	Last 2 years	Cases more likely to have a long-term sick leave (> 14 days)	_
[61]	E	Bladder pain	C/A	No	No	48	Last week	Cases with a higher proportion of missed work time	_
								Cases with a higher proportion of perceived impairment at work	

Abbreviations. Ref. Reference, NA: North America, E: Europe, O: other, C: Childhood, C/A: Childhood/Adulthood, CI: FU: Follow-up, Y/N: Yes/No, y.: year

^*^ Mean age at study or most prevalent age group of cases

**i) Absenteeism**, primarily measured by the number of sick leave days or the percentage of work time missed due to health issues, demonstrated at least one significant difference in 90% of the studies (n = 18/20) (Table 2). Results were less likely to be significant when the outcome was a continuous variable (e.g., number of hours of missed work) compared to a dichotomized variable (yes/no). Notably, in two studies, three outcomes initially showed no significant differences but became significant only two or five years after diagnosis or throughout the entire follow-up period [35, 56] (Table 4).

**ii) Presenteeism** was explored in 6 studies primarily through the impairment experienced at work due to health, whether through a dichotomized outcome or outcomes measuring the proportion of impairment or work capacity. Overall, work impairment was significantly worse for individuals with rare diseases across 100% of studies (n = 6/6) (Tables 2 and 4).

**iii) Work loss**, which combines days of disability and days of sick leave recorded in administrative databases, was found to be worse for patients in 100% of studies that included this outcome (n = 5/5) (Tables 2 and 4).

In summary, employed individuals with rare diseases generally experienced significantly more sick leave days, missed more work time than did controls and felt more impaired at work (Table 2).

## Discussion

This systematic review, encompassing 44 peer-reviewed articles, provides valuable insights into how rare diseases are associated with individuals’ employment status and work ability, trying to acknowledge the less-explored health selection effects. Indeed, 87% of studies found that individuals with rare diseases were more likely to be work disabled than controls, experienced more absenteeism (90% of studies), or experienced more impairment at work (100% of studies). These findings underscore a general hindrance to employment and work ability posed by rare diseases. In particular, work ability was deteriorated in almost all studies, irrespective of the disease characteristics (such as physical or cognitive limitations, organ/system affected, or timing of onset).

Regarding employment, the associations of rare diseases with disability and health-related unemployment are in line with other studies showing a greater disability rate in individuals with chronic diseases such as cancer, cardiovascular diseases, diabetes, lung disease, or arthritis [66–68]. In this review, the timing of onset of the rare disease was not found to be of particular importance since almost all studies which included this outcome found a higher disability rate in patients. This contrasts with frequent chronic diseases, where age at diagnosis and illness duration are associated with chances to participate in the labour market [69]. However, given the small number of studies with a childhood onset included in this literature review, further investigation into the impact of age at onset across a broader range of diseases might be warranted.

Regarding work ability or work time, although individuals with rare diseases had higher disability rates than controls in the included studies, a majority of working-age adults with rare diseases were employed. Almost all studies, whether they evaluated absenteeism several years after diagnosis or shortly after diagnosis, reported a worse situation for patients, probably reflecting the impact of physical and possibly psychological symptoms on work ability. Moreover, employed patients with rare diseases were more likely to work part-time than controls [26, 32, 47], possibly because of the time required for medical follow-up and/or the limitations associated with the disease. These patterns of absenteeism or part-time employment are also found in individuals with frequent chronic diseases [66, 70].

The results of this literature review tend to show an impact of rare diseases on work, illustrating health selection effects. Indeed, disability or health-related unemployment significantly contributes to socioeconomic poverty and increases the probability of downward mobility [71]. In addition, both absenteeism and part-time work may result in lower income and capital accumulation [72]. Yet, the methodological limitations of the included studies hamper the understanding of health selection effects. Indeed, since most rare diseases are genetic and have a paediatric onset, the direction of causality is more likely to be unique, with the disease negatively influencing work-related outcomes. However, some rare diseases involve epigenetic pathways [73] and/or can be related to occupational exposure, especially rare lung diseases. Reverse causality is thus possible, with inequalities in incidence leading disadvantaged populations to be overrepresented in cases, who may subsequently be more likely to have a poor work situation because of their socioeconomic background. In this review, 39% (n = 17) of the studies included diseases potentially related to occupational exposure or environmental factors, and only 32% (n = 14) were conducted on paediatric-onset diseases. This result is likely influenced by challenges in identifying adults with rare diseases that manifest in childhood. The reasons for this are twofold. First, in studies originating from clinical settings or rare disease registries, approximately half of the patients may be lost to follow-up after transitioning to adult care [74]. This is a significant barrier to identifying these individuals in adulthood. Second, general registries are often reliant on the International Classification of Diseases (ICD) system. The ICD-10 provides specific codes for only a limited number of rare diseases (approximately 5%) [75], limiting their identification in such databases. Rare diseases linked to occupational exposure, which are more likely to occur in adulthood, may be easier to identify due to administrative requirements associated with these conditions and may explain the high proportion of diseases with an adult onset potentially related to occupational exposure or environmental factors in our literature review. An adjustment for education could help in the measurement of the impact of rare diseases on work, but only 5 studies controlled for the effects of education on labour force participation [23, 53, 60, 63, 65], thus reducing our understanding of health selection effects. Furthermore, as time passes, individuals with rare diseases may become less able to cope with the demands of the job, and this may be compounded by the time-consuming use of medical services and frequent sick leaves, especially for patients in physical occupations. Only one study matched cases and controls on the type of occupation [52], limiting the understanding of the interplay between health and social inequalities [76]. Finally, impairment at work, absenteeism or part-time work may negatively impact career development, but only a few studies included in this literature review used a longitudinal design [24, 28, 35, 45] and none of them investigated the effects of having a rare disease over the life course to show the potential relationships between absenteeism/presenteeism and subsequent exit from the labour force.

While individuals with rare diseases experience employment challenges, these are probably mediated by disease-specific characteristics, in particular for individuals with significant cognitive impairments, who may face greater challenges. Other research, even if not including a longitudinal follow-up or a case–control design, provided some fruitful insights into the determinants of work participation of rare disease patients, highlighting the factors negatively associated with employment, including disease-related factors (such as disease severity, fatigue, pain, depression, and reduced quality of life) or social factors (lower education levels, higher age) [8]. Disease-related factors can for instance explain the poorer outcomes of patients with SLE compared to outcomes of patients with JIA highlighted in the results of our literature review. Indeed, SLE is a chronic autoimmune disease that often involves multiple organs, including the kidneys, heart, and central nervous system with significant physical limitations and reduced health-related quality of life [77] making consistent employment more challenging. While JIA can cause joint inflammation and damage, long-term remission or low disease activity is more achievable in JIA [78], which may reduce its impact on employment outcomes. On the other hand, the study with an opposite result showing more employed female patients with Turner Syndrome may actually reflect a positive impact of the disease on work attainment, potentially due to the fact that, in this study, women with Turner Syndrome were less likely to marry and have a family. These factors significantly impact work ability among women, as family responsibilities and caregiving roles can influence employment choices, working hours, and career progression. The reduced likelihood of family-related obligations in this population may enable higher rates of participation in the workforce, potentially contributing to the observed employment advantage [58].

It is important to highlight other methodological pitfalls of the included studies for future research. Indeed, the absence of findings (i.e., studies not showing a poorer situation for cases) seems to be related to methodological aspects, with nonsignificant results mainly found in studies on young adults [26, 51] or with a small sample size [34, 40, 57]. In addition, nine studies were based on data from questionnaires completed by patients from a single institution, with possible cumulative bias related to the recruitment of patients and their participation in a questionnaire study [79].

Future research perspectives can be derived from this literature review. First, studies on work-related outcomes should exclude young adults (< 25 years old) who may not have finished education to show the effects of a given disease on work. Secondly, a large set of studies was excluded because they were only descriptive and failed to include an analytic approach (e.g., no control group or no appropriate statistical analysis). A matched control group is essential to accurately establish health selection effects, as it allows for a direct comparison between individuals with a specific condition and those without it while controlling for confounding variables such as age, gender, education, occupation and socioeconomic status, or comorbidities. By matching the control group to the patient group, researchers can isolate the impact of the disease itself on employment outcomes, rather than attributing observed differences to unrelated demographic, social or medical factors. Given the importance of comorbidities in rare diseases, an adjustment is very important to understand the specific effect of a given rare disease, while the exclusion of some conditions in controls makes the interpretation of results complex and hampers comparisons between studies. Furthermore, only one study included analyses adjusted for a psychiatric diagnosis, although there is a known association between unemployment and mood-affective disorders such as depression, which is highly correlated with absenteeism in the general population [80] and which can be frequent in individuals living with rare diseases [81]. Most importantly, comparisons of cases and controls should include social factors such as education or occupation to avoid methodological bias since employment-related outcomes are closely related to these factors in the general population [82]. Third, longitudinal research controlling for medical or social characteristics should be carried out to investigate more rigorously health selection effects, whether they follow one outcome over time (e.g., employment), or whether they try to measure the effect of absenteeism on the employment or social mobility of patients. In addition, employment or unemployment needs to be clearly defined and calculated, using international classifications or those used in international surveys to facilitate comparisons. Indeed, studies investigating “unemployment” without making a distinction between the different reasons for unemployment (i.e., including in the same group homemakers, students, individuals actively seeking work, and those unable to work because of health) can mask significant differences. Other research designs, such as descriptive cross-sectional studies, longitudinal studies, and qualitative in-depth studies, are also crucial for advancing our understanding of the impact of rare diseases on work. Descriptive and multivariate analyses can offer important baseline data, while longitudinal studies can help establish causal relationships and evaluate interventions. Furthermore, qualitative research that captures patients' own experiences and perceptions provides valuable insights into the lived realities of individuals with rare diseases, complementing quantitative findings.

Finally, contextual elements such as welfare system organization and cultural norms play a significant role in determining employment outcomes for individuals with disabilities, including those with rare diseases. Employment rates among individuals with disabilities vary across regions [83], with different cultural and societal attitudes towards disability and policy frameworks potentially influencing work outcomes. For example, countries with a robust welfare system and greater cultural acceptance may provide more comprehensive support for individuals with rare diseases, facilitating higher employment rates. Conversely, in regions where such support is less structured or with less inclusive policies, employment participation may be lower. In future research, it would be valuable to explore how these contextual factors interact with other dimensions to shape employment outcomes.

### Strengths and limitations

This literature review includes 44 studies and provides, to our knowledge, an important overview of the effects of rare diseases on work at the individual level in studies with robust methods. We aimed to ensure a rigorous systematic review by following the PRISMA checklist [17]. In the three electronic databases searched, we included the names of 695 rare diseases with a point prevalence or annual incidence > 1/100,000, as the probability of finding eligible studies was greater than for diseases with a lower incidence/prevalence. Although generic keywords related to rare diseases (e.g., “rare”) were included in the search strategy, we acknowledge the possibility of missing some publications. The selection process for eligible studies and data extraction was conducted by two independent reviewers, with the consultation of a third independent reviewer to resolve any conflicts, minimizing potential biases related to the selection of articles.

A limitation of this systematic review is the generalizability of the results reported in the included studies. Only studies written in English and published between 2013 and 2023 were examined. Most of the studies were conducted in Europe and North America, limiting the generalizability of the findings to high-income countries. Furthermore, this literature review included only 34 rare diseases, which is little considering the 6000 rare diseases registered in databases such as Orphanet [3] or the 695 rare diseases with a point prevalence or annual incidence > 1/100,000 included in the search strategy. Yet, this little number of rare diseases is comparable to the one found in a scoping review on work participation in adults with genetic rare diseases, which found articles on 33 rare diseases solely, even if published from 2000 on and using very different designs, including qualitative studies [8]. This limitation may reflect the difficulties of gathering enough cases to conduct quantitative studies for numerous rare diseases and emphasizes the need to investigate a broader range of rare diseases to ensure a comprehensive understanding of employment-related outcomes in this population. In addition, in most of the studies, patients were diagnosed in adulthood, which does not reflect the epidemiology of rare diseases. The skewed selection of diseases may limit the generalizability of our findings. This limitation is likely due to challenges in identifying adults with childhood-onset rare diseases, particularly in clinical settings and registry studies, as well as the constraints of the ICD system.

We did not explore the underlying mechanisms of unemployment or work inability, particularly in terms of comorbidities, psychological burden associated with the disease, or societal norms of the firms, since this information was not consistently provided in the studies. Only half of studies matched cases and controls using social variables such as education, which is a limit of studies potentially limiting the results of this literature review. We chose to highlight the existence and significance of differences between cases and controls rather than providing exact proportions and results of statistical analyses, given the heterogeneity in study design and sampling across studies, which also hampered the conduct of a meta-analysis. Finally, we used and adapted the Newcastle‒Ottawa Scale for case‒control studies [21]. Even if the NOS score cannot be interpreted because of this adaptation, almost one third (32%) of studies scored below six. However, this scale does not consider the sample size of the studies, which may be small due to the rare nature of the disease. To address this issue, we independently considered the sample size of studies when interpreting the results.

## Conclusion

Patients with rare diseases often have lower employment rates and especially greater disability rates than controls. Moreover, even among those individuals with rare diseases who are employed, there are significant poorer results for both absenteeism and presenteeism. No clear patterns were detected concerning diseases with cognitive and mobility impairments, the timing of onset, or whether the rare disease was of systemic or organ origin regarding work ability. This suggests that rare diseases distinctly hamper work ability, regardless of the type of limitations, whether physical or cognitive. Additionally, the labour market challenges faced by individuals with rare diseases may vary depending on contextual factors at the meso (firms) or macro (national work policies) levels, highlighting the complexity of their employment experiences. Working conditions, such as remote working or policies on the protection of people with poor health at the macro level, could either facilitate or hamper the chances of remaining employed, thereby limiting the social inequalities generated by rare diseases. Besides, while many measures for chronic diseases could be applicable to rare disease patients, specific interventions at different levels may be needed because of the young age at diagnosis in many rare diseases: at the macro level, through a recognition of the specific situation of individuals with rare diseases in employment programmes and disability frameworks; at the organizational level as flexible work arrangements may need adjustments to account for the specific challenges of rare diseases, such as cognitive impairment, fluctuating symptoms or limited healthcare access; and at the micro-level, considering the variability in symptoms and treatment, personalized healthcare, career counselling, and targeted support are essential for individuals with rare diseases. National and European policymakers and health planners should consider these aspects when designing strategies, policies, and plans to achieve comprehensive care and equity for affected individuals. Finally, clinicians, employers and policy makers should be made aware of the potential impact rare diseases can have on patients' work capacities to better address their work-related needs. This includes recognizing how patients' ability to sustain employment can be hampered by disease-related factors—such as early onset, multisystem involvement and symptoms—, healthcare related factors—such as the impact of the time required for follow-up— and societal factors—such as norms towards disability. Understanding all these nuances is crucial to design targeted interventions and policies that support work participation among individuals with rare diseases.

## Supplementary Information

Additional file1 (DOCX 29 KB)

## Data Availability

Not applicable since all data analysed in this study are secondary data from publicly available manuscripts.
