# Supplementary material for: Employment and work ability in individuals living with rare diseases: a systematic literature review
Source: Orphanet J Rare Dis. 2025 Apr 23;20:193. doi: 10.1186/s13023-025-03691-7 (PMC12020228; doi:10.1186/s13023-025-03691-7)
Supplement: Supplementary file 1 — Additional file1 (DOCX 29 KB) [file 13023_2025_3691_MOESM1_ESM.docx]

Supplemental Table S1. Search Query

|  | Pubmed | Embase | Web of Science |
| --- | --- | --- | --- |
| Rare diseases terms | ("Orphanet Journal of Rare Diseases"[Journal] OR "Rare Diseases"[Mesh] OR ((“rare”[tw] OR “orphan”[tw]) AND (“diseas*”[tw] OR “illness*”[tw] OR “condition*”[tw] OR “disorder*”[tw]))) | ('orphanet j. rare dis.':ta OR ‘rare Disease’/de OR (('rare' OR 'orphan') AND ('diseas*' OR 'illness*' OR 'condition*' OR 'disorder*'))) | (SO=(ORPHANET JOURNAL OF RARE DISEASES) OR ((ALL=(rare) OR ALL=(orphan)) AND (ALL=(diseas*) OR ALL=(illness*) OR ALL=( condition*) OR ALL=( disorder*)))) |
| Names and synonyms of 695 rare diseases | Excel file containing Pubmed's list of rare diseases names/synomyms with point prevalence or annual incidence >1/100,000 (World, Europe, US) | Excel file containing Embase's list of rare diseases names/synomyms with point prevalence or annual incidence >1/100,000 (World, Europe, US) | Excel file containing Web of Science's list of rare diseases names/synomyms with point prevalence or annual incidence >1/100,000 (World, Europe, US) |
| Work-related terms | ("Work"[Mesh:NoExp] OR "Employment"[Mesh:NoExp] OR "Employment"[tiab] OR "Employment, Supported"[Mesh] OR "Unemployment"[Mesh] OR "Unemploy*"[tiab] OR "Jobless"[tiab] OR "Return to Work"[Mesh] OR "Return to Work"[tiab] OR "Absenteeism"[Mesh] OR "Presenteeism"[Mesh] OR "Absenteeism"[tiab] OR "Presenteeism"[tiab] OR "missing work"[tiab] OR "Sick Leave"[Mesh] OR "Sick Leave"[tiab] OR "medical leave"[tiab] OR "Work ability"[tiab] OR "Work disability"[tiab] OR "Work performance"[Mesh] OR "Work productivity"[tiab] OR "Workload"[Mesh]) | (‘Work’/de OR 'employment'/de OR ‘Employment’:ti,ab OR ‘supported employment’/exp OR ‘Unemployment’/exp OR ‘Unemploy*’:ti,ab OR ‘Jobless’:ti,ab OR ‘Return to Work’/exp OR ‘Return to Work’:ti,ab OR ‘Absenteeism’:ti,ab OR ‘Absenteeism’/exp OR ‘Presenteeism’/exp OR ‘Presenteeism’:ti,ab OR ‘missing work’:ti,ab OR ‘Sick Leave’:ti,ab OR ‘medical leave’/exp OR ‘medical leave’:ti,ab OR ‘Work ability’:ti,ab OR ‘Work disability’:ti,ab OR ‘ Job performance’/exp OR ‘Work productivity’:ti,ab OR ‘ ‘Workload’/exp) | (TS=(Employment) OR TS=(Unemploy*) OR TS=(Jobless) OR TS=(“Return to Work”) OR TS=(Absenteeism) OR TS=(Presenteeism) OR TS=("missing work") OR TS=("Sick Leave") OR TS=("medical leave") OR TS=(“Work ability”) OR TS=(“Work disability”) OR TS=(“Work productivity”)) |
| Excluded terms | NOT "Occupational Exposure"[Mesh] NOT "Occupational Diseases"[Mesh] NOT "Occupational Health"[Mesh] NOT "occupational expos*"[tiab] NOT "occupational therap*"[tiab]) NOT treatment*[title] NOT drug*[title] NOT medication*[title] NOT protocol*[title] NOT “randomised controlled trial”[title] NOT “randomized controlled trial”[title] NOT RCT[title] NOT prevention*[title] NOT recommendation*[title] NOT exposure*[title] NOT “social determinant*”[title] NOT “clinical determinant*”[title] NOT COVID19[title] NOT COVID 19[title] | NOT ‘Occupational Exposure’/exp NOT ‘Occupational Disease’/exp NOT ‘Occupational Health’/exp NOT ‘occupational expos*’:ti,ab NOT ‘occupational therap*’:ti,ab NOT ‘treatment*’:ti NOT ‘drug*’:ti NOT ‘medication*’:ti NOT ‘protocol*’:ti NOT ‘randomi?ed controlled trial’:ti NOT ‘RCT’:ti NOT ‘prevention*’:ti NOT ‘recommendation*’:ti NOT ‘exposure*’:ti NOT ‘social determinant*’:ti NOT ‘clinical determinant*’:ti NOT ‘COVID19’:ti NOT ‘COVID 19’:ti | NOT TS=("occupational expos*") NOT TS=("occupational therap*") NOT TI=(treatment*) NOT TI=(drug*) NOT TI=(medication*) NOT TI=(protocol*) NOT TI=( “randomi?ed controlled trial”) NOT TI=(RCT) NOT TI=(prevention*) NOT TI=(recommendation*) NOT TI=(exposure*) NOT TI=(“social determinant*”) NOT TI=(“clinical determinant*”) NOT TI=(COVID19) NOT TI=(“COVID 19”) |
| LANGUAGE AND DATE OF PUBLICATION | (English[lang]) AND (2013:2023[pdat]) | [english]/lim AND [2013-2023]/py AND [article]/lim | LA=(English) AND PY=(2013-2023) AND DT=(Article) |

Supplemental Table S2. Overview and details of included studies

| **Ref.** | **Country** | **Year** | **Name of the disease(s)** | **Main organ/system/ function affected** | **Timing of onset** | **Number of patients** | **Indentification of cases (and controls if different from cases)** | **Method of comparison with controls** | **Medical characteristics of controls*** | **Social characteristics of controls**** | **Type(s) of work-related outcomes** | **Source of data collection for work-related variables in cases (and for controls if different)** |
| --- | --- | --- | --- | --- | --- | --- | --- | --- | --- | --- | --- | --- |
| [20] | Sweden | 2018 | Myasthenia gravis (MG) | Muscles | C/A | 40 | Multicentre Study (National Administrative Registry) | Matching | ERD | *Region of Residence* | Work ability | Questionnaire |
| [21] | Norway | 2020 | Acute hepatic porphyria (AHP) | Liver | C/A | 293 | National RD Registry (National Administrative Registry) | Matching | ERD | *Education* | Employment, work ability | Questionnaire |
| [22] | Denmark | 2019 | Sarcoidosis | Systemic | C/A | 9119 | National Administrative Registry | Matching | ERD | *Region of Residence* | Employment | Administrative data |
| [23] | Australia | 2015 | Childhood onset multiple pituitary hormone deficiencies (COMPHD) | Endocrinal | Childhood | 92 | Multicentre Study (Siblings, friends or volunteers) | Matching | Self-reported as healthy | None | Employment | Questionnaire |
| [24] | USA | 2015 | Systemic lupus erythematosus (SLE) | Systemic | C/A | 344 | Multicentre Study (Friends of cases) | Chi-square comparison | ERD | Sibling/friends recruitment | Employment, work ability | Questionnaire |
| [25] | USA | 2014 | Narcolepsy | Sleep | C/A | 600 | Health Claims Database | Matching | ERD | *Type of Insurance (Medicare or not)* | Employment | Administrative data |
| [26] | Sweden | 2018 | Sarcoidosis | Systemic | C/A | 3347 | National Administrative Registry | Matching | ERD | *Place of Residence* | Work ability | Administrative data |
| [27] | USA | 2016 | Dermatomyositis, Polymyositis | Muscles | C/A | 2587 | Health Claims Database | Matching | ERD, **CCI**, **Other** | None | Employment, work ability | Administrative data |
| [28] | Canada | 2019 | Idiopathic lung disease, Idiopathic nonspecific interstitial pneumonia, Chronic hyper-sensitivity pneumonitis | Lung | C/A | 650 | Multicentre RD Registry (National Statistics) | Standardization | None | None | Employment | Questionnaire  (National Statistics) |
| [29] | USA | 2019 | Systemic sclerosis | Systemic | C/A | 233 | Health Claims Database | Matching | ERD | *Insurance type, Region of Residence* | Employment, work ability | Administrative data |
| [30] | France | 2022 | Haemophilia | Blood | Birth | 588 | Single Centre Study (National Statistics) | Standardization | None | None | Employment | Questionnaire,  National Statistics |
| [31] | Canada | 2020 | Fibrotic CTD-ILD | Lung | C/A | 375 | National RD Registry (National Statistics) | Standardization | None | None | Employment | Questionnaire,  National Statistics |
| [32] | USA | 2017 | Juvenile idiopathic arthritis (JIA) | Joints | Childhood | 38 | Local Administrative Registry | Matching | ERD | *Region of Residence* | Employment | Administrative data |
| [33] | Sweden | 2017 | Sjogren’s syndrome | Systemic | C/A | 51 | Single Centre Registry (National Administrative Registry) | Matching | None | *Place of Residence* | Employment, work ability | Administrative data |
| [34] | Sweden | 2021 | Sjogren’s syndrome | Systemic | C/A | 8884 | National Administrative Registry | Matching | ERD | *Region of Residence* | Employment | Administrative data |
| [35] | Germany | 2017 | Juvenile idiopathic arthritis (JIA) | Joints | Childhood | 2183 | Single Centre Study (National statistics) | Standardization | None | None | Employment | Questionnaire,  National Statistics |
| [36] | 6 European countries~~*~~ | 2021 | Turner syndrome | Systemic | Birth | 328 | Multicentre Study (National Survey) | Matching | None | None | Employment | Questionnaire |
| [37] | Germany | 2017 | Non-tuberculous mycobacterial pulmonary disease | Lung | C/A | 125 | Health Claims Database | Matching | ERD, **CCI** | None | Work ability | Administrative data |
| [38] | Canada | 2019 | Systemic lupus erythematosus, systemic sclerosis, Sjögren's syndrome | Systemic | C/A | 299 | Local Administrative Registry | Matching | ERD | None | Employment, work ability | Questionnaire |
| [39] | Finland | 2018 | Systemic Lupus Erythematosus (SLE) | Systemic | C/A | 446 | National Administrative Registry | Standardization | None | None | Employment | Administrative data |
| [40] | Turkey | 2021 | Familial Mediterranean fever | Systemic | C/A | 111 | Single Centre Study (Not stated) | Matching | Other | None | Work ability | Questionnaire |
| [41] | Turkey | 2022 | Retinitis Pigmentosa | Eyes | C/A | 70 | Single Centre Study (Not stated) | Matching | Other | None | Employment | Questionnaire |
| [42] | Denmark | 2021 | Idiopathic/genetic generalized epilepsies (IGEs) | Neurological | Childhood | 167 | Multicentre study (Local administrative registry) | Matching | ERD | *Municipality of Residence* | Employment | Questionnaire |
| [43] | Denmark | 2016 | Myasthenia gravis (MG) | Muscles | C/A | 330 | National Administrative Registry | Matching | ERD | *Occupation* | Employment, work ability | Administrative data |
| [44] | Finland | 2015 | Systemic Lupus Erythematosus (SLE) | Systemic | C/A | 181 | Single Centre Study (National Survey) | Matching | None | *Municipality of residence* | Employment, work ability | Questionnaire |
| [45] | Netherlands | 2021 | Systemic Lupus Erythematosus (SLE) | Systemic | C/A | 106 | Multicentre Study (National Statistics) | Standardization | None | None | Employment | Questionnaire,  National Statistics |
| [46] | Denmark | 2016 | Turner Syndrome | Systemic | Birth | 56 | Single Centre registry (National Administrative Registry) | Matching | None | *Place of Residence* | Employment | Questionnaire |
| [47] | Germany | 2019 | Bronchiectasis | Lung | C/A | 231 | Health Claims Database | Matching | ERD, **CCI** | None | Work ability | Administrative data |
| [48] | USA | 2022 | Pulmonary arterial hypertension (PAH) | Cardiovascular | C/A | 1293 | Health Claims Database | Matching | ERD | *Region of Residence* | Work ability | Administrative data |
| [49] | Denmark | 2020 | Narcolepsy | Sleep | Childhood | 171 | National Administrative Registry | Matching | ERD | *Municipality of residence* | Employment | Administrative data |
| [50] | USA | 2021 | Acromegaly | Endocrinal | C/A | 47 | Health Claims Database | Matching | ERD | *Race, Job-related variables* | Employment, work ability | Administrative data |
| [51] | Denmark | 2023 | Haemophilia | Blood | Birth | 124 | Multicentre study (National Survey) | Adjusted regression | None | Education | Employment | Questionnaire |
| [52] | Denmark | 2019 | Atrial Septal Defect | Cardiovascular | Birth | 2277 | National Administrative Registry | Matching | ERD, Other | None | Employment, work ability | Administrative data |
| [53] | Sweden | 2023 | Pulmonary arterial hypertension (PAH) | Cardiovascular | C/A | *749* | National Administrative Registry | Matching | ERD | *Municipality of residence* | Employment, work ability | Administrative data |
| [54] | USA | 2016 | Non infectious posterior uveitis | Eyes | C/A | 776 | Health Claims Database | Matching | ERD, **CCI**, Other | *Region of Residence* | Employment, work ability | Administrative data |
| [55] | Sweden | 2014 | Crouzon syndrome | Skull | Birth | 31 | Single Centre registry (National Administrative Registry) | Adjusted regression | None | Region of Residence | Employment | Questionnaire |
| [56] | USA | 2013 | Turner syndrome | Systemic | Birth | 240 | Single Centre Study (National Statistics) | Standardization | None | None | Employment | Questionnaire,  National Statistics |
| [57] | UK, Ireland | 2022 | Juvenile Dermatomyositis | Skin | Childhood | 84 | Multicentre RD Registry (National statistics) | Standardization | None | None | Employment | Questionnaire,  National Statistics |
| [58] | USA | 2016 | Narcolepsy | Sleep | C/A | 437 | National Survey | Matching | ERD, **CCI** | *Income, Education, Race* | Employment, work ability | Questionnaire |
| [59] | Denmark | 2014 | Hypersomnia | Sleep | C/A | 2855 | National Administrative Registry | Matching | ERD | *Region of Residence* | Employment | Administrative data |
| [60] | Sweden | 2014 | Congenital adrenal hyperplasia (CAH) | Endocrinal | Birth | 588 | National Administrative Registry | Matching | ERD | *Place of birth* | Employment, work ability | Administrative data |
| [61] | 5 European countries~~**~~ | 2017 | Bladder Pain | Bladder | C/A | 275 | National Survey | Matching | ERD, **CCI** | *Employment status, Income, Education* | Employment, work ability | Questionnaire |
| [62] | Denmark | 2015 | Meningococcal meningitis | Neurological | C/A | 1804 | National Administrative Registry | Matching | ERD, Other | Siblings | Employment | Administrative data |
| [63] | United Kingdom | 2022 | Aneurysmal Subarachnoid Haemorrhage | Brain | C/A | 884 | General population cohort | Matching | ERD, Other | *Deprivation Index, Education* | Employment | Questionnaire |

Abbreviations. Ref. Reference, RD: Rare disease; C/A: Childhood/Adulthood; UK: United Kingdom; ERD: Exclusion of the Rare Disease under investigation; CCI: Matching on the Charlson Comorbidity Index; NOS: Newcastle-Ottawa Score

* Medical variables used for matching are in bold text, medical variables used for the selection of the study population or other statistical techniques are not in bold text

** Social variables used for matching are in bold text, social variables used with other statistical techniques are not in bold text
